# The illusion of inclusion: examining the limitations of diversity metrics in baseball

**DOI:** 10.3389/fpsyg.2025.1512033

**Published:** 2025-03-26

**Authors:** Claire Sandman Malcomb, Emily Zitek

**Affiliations:** School of Industrial and Labor Relations, Cornell University, Ithaca, NY, United States

**Keywords:** diversity equity and inclusion, diversity metrics, sports, Major League Baseball, measuring diversity, Latin American players and coaches

## Abstract

The importance of diversity, equity, and inclusion (DEI) in sports has become widely discussed in recent years, and many sports organizations have dedicated extensive resources to DEI initiatives. Much of the focus has been on the representation of underrepresented minorities within sports, and many organizations rely heavily on representation as a metric for success of their DEI initiatives. In the current work, we examine the potential limitations of diversity metrics currently used in professional sports organizations. Recently, reports have suggested that player and coach diversity in Major League Baseball are good, with high international Latino representation, and DEI efforts should therefore be focused elsewhere. However, across two studies utilizing datasets of Major and Minor League Baseball players (Study 1) and coaches (Study 2), we find evidence that, despite the high diversity, some baseball players and coaches in the lower levels seem to have less ability to advance than others. Specifically, international Latino players are less likely to make it to as high of a level in baseball as US-born players, and international Latino coaches are more likely to get stuck in the lower levels of coaching. Moreover, in a third study surveying players in the independent Frontier League, we find evidence that non-White players feel less of a sense of inclusion. We provide insight and recommendations for how sports organizations and researchers can better measure and interpret the progress of DEI initiatives in sports.

## 1 Introduction

A great deal of work has documented the organizational advantages of diversity, equity, and inclusion (DEI) initiatives, defined as practices or policies geared toward improving the experiences and outcomes of people from underrepresented and historically marginalized identity groups (Dobbin and Kalev, 2022; Nishii et al., 2018; Nkomo et al., 2019; Portocarrero and Carter, 2022; Roberson, 2019). Generally, DEI initiatives aim to (1) increase the representation of underrepresented minorities, (2) reduce gaps in success between marginalized and non-marginalized groups, and (3) develop more inclusive work environments (Leslie, 2019). Increasing an organization’s diversity—often understood as the numerical representation of people from underrepresented identity groups—is the most used metric for the success of DEI initiatives (Leslie, 2019). While increasing the representation of people from underrepresented and marginalized communities is important, overreliance on diversity numbers as a sole measure of the success of DEI initiatives may give a false sense of progress (Leslie, 2019; Nishii et al., 2018; Nkomo et al., 2019). Further, as organizations become more diverse, it is important that they are also intentional in their efforts to create more inclusive environments, which can produce job satisfaction, organizational commitment, and decreased stress (Shore et al., 2018). Recognizing the importance of inclusion, there have been calls to move beyond efforts and metrics focused solely on diversity to work that deepens our understanding of how to create truly inclusive organizations in which everyone is treated fairly, is valued for who they are, feels that they belong, and can fully engage at all levels within the organization (Nishii et al., 2018; Nkomo et al., 2019; Roberson, 2019; Shore et al., 2018). Importantly, this shift toward expanding the scope of how we measure the success of DEI initiatives adds to, rather than replaces, the importance of increasing representation of minorities within organizations, especially in high-level positions.

Given that minority groups are often underrepresented in organizations, especially at higher levels within the organization, there are few contexts in which it is possible to empirically examine or measure the additional barriers to inclusion in highly diverse organizations. However, sports organizations tend to have a higher representation of minority group members as compared to other organizational domains (e.g., African-Americans are well represented as players in many professional sports such as football and basketball; Czopp et al., 2015; Kay et al., 2013). Given this, the sports domain is uniquely positioned for scholars to examine the additional barriers to inclusion that exist for minorities even in highly diverse organizations. In fact, sports have been widely used as a context to study the treatment of minority groups (Carrington, 2015; Ertug and Maoret, 2020; Lowe, 2021; Parsons et al., 2011; Sartore and Cunningham, 2006; Zhang, 2017).

In the current work we examine Major League Baseball (MLB) as a case study of how the most common DEI metric, the representation of historically underrepresented minorities, can create inflated perceptions of progress that are counterproductive to positive change. Importantly, we do not argue that MLB and other organizations should not utilize diversity numbers as a metric of the effectiveness of DEI initiatives. Rather, we examine evidence of the limitations of diversity as a sole metric and explore how metrics of gaps in success and perceptions of inclusion can provide important insights for sports leagues and organizations as they make policy decisions.

### 1.1 Measuring diversity in sports

The Institute for Diversity and Ethics in Sports (TIDES) conducts research and publishes reports related to gender and racial equity and ethical issues in collegiate and professional sports. One of the main reports TIDES publishes is an annual Racial and Gender Report Card for various professional sports leagues such as Major League Baseball (MLB). The Report Cards include information on hiring practices, demographic composition of the league at every level (i.e., players, coaches, managers, owners, etc.), and other relevant information about a league’s diversity practices. These Racial and Gender Report Cards are internationally recognized and provide important information to sports leagues as they aim to develop DEI initiatives.

Looking at baseball specifically, in 2023 MLB received an A for player racial diversity and an A+ for coach racial diversity on their Report Card, with 40.3% of players and 38.3% of coaches in MLB identified as “people of color,” many of whom are international Latinos (Lapchick, 2023a). Like other sports organizations, MLB’s racial diversity quickly declines when looking at the front offices, receiving only a D+ for presidents/GMs and a C+ for senior administration. Thus, a tempting interpretation of the MLB Report Card is that racial diversity on the field is acceptable, and DEI initiatives should focus on (1) gender diversity among coaches and (2) racial and gender diversity in the front offices [see Lapchick (2023b)]^1^. To be clear, both of these goals are important and warrant attention. However, it is also important to understand whether the high racial diversity reported at the player and coaching levels indicates that DEI goals have been achieved on the field and thus warrants “moving on” to focus efforts on other areas. It is possible that the high diversity grades do not necessarily mean that baseball is completely inclusive and fair for all players and coaches. In line with this, interviews we conducted with Minor League Baseball (MiLB) players revealed that international Latino players may face additional barriers, such as language barriers and cultural differences, that make it harder for them to advance through the professional baseball system (Malcomb et al., in press).

### 1.2 Overview of current research

In the current work, we aimed to reconcile the high diversity grades for players and coaches (Lapchick, 2023a) with other evidence of additional barriers to advancement in baseball for international Latino players (Malcomb et al., in press). We first wanted to understand whether the diversity reports showing that MLB has high representation at the top playing level painted a complete picture of how inclusive and fair the whole baseball system is. Though the TIDES reports only utilize data from the Majors, MiLB is part of the MLB system; when players are drafted or signed by a team, they usually start at a low level in the Minors and get moved up to a higher level as they improve. Therefore, in Study 1, we utilized an archival dataset of baseball players in the Majors and Minors to examine whether international Latino players and players from other underrepresented groups displayed evidence of less career progression. In Study 2, we created a cross-sectional dataset of coaches across all levels of the Minors and Majors to examine whether international Latino coaches were less likely to move up in the ranks. In Study 3, we aimed to supplement our findings from the first two studies by collecting survey data from players in the Frontier League related to their perceptions of inclusion in baseball. Overall, we hope our work will help organizations more effectively utilize DEI metrics as tools to advance diversity, equity, and inclusion.

## 2 Study 1

### 2.1 Method

We developed a dataset of professional baseball players in MiLB and MLB by merging data from two sources. Our first source was data from Baseball Cube, a reputable repository of baseball statistics and information (Baseball Cube, 2025). This dataset consisted of all players who played in MiLB and/or MLB from 1992 (the first year of the 7-tiered system for progression from the Minors to the Majors; Baseball Reference, 2023a) through 2022. We merged these data with self-reported player ethnicity data collected by MLB and shared with our research team. Our final dataset comprised 50,477 players (30,343 were US-born, and 18,404 were born in a Latin American country/territory), but we used different subsets of players for the different analyses. Please see the Supplementary materials (SOM) for additional information about our methods. We had the following two primary outcome variables of interest.

#### 2.1.1 Highest level achieved

This dependent variable captures the highest level in MiLB or MLB that a player achieved in their career, from Rookie Ball (Rk), Short Season A ball (SS), A, Advanced A, AA, AAA, and MLB (the 7-tiered system; Moore, 2013). These were coded 1–7 with 1 being the lowest level (Rk) and 7 being the highest level (MLB).^2^

#### 2.1.2 Longevity

This dependent variable captures the number of years each player spent in baseball at any level, up through 2022. It was calculated by subtracting the start year from the end year for each player and adding 1.

### 2.2 Results

To examine differences in the progression through baseball between US-born players and international Latino players, we ran independent-samples *t* tests predicting highest level achieved and longevity from nationality. We found that US-born players made it to significantly higher levels within baseball during their career compared to international Latino players, advancing about a level higher on average (see Table 1). Moreover, the US-born players also had significantly greater longevity than the international Latino players, playing baseball for almost two years longer on average (see Table 1). Given that making it to MLB is the ultimate goal, we also examined how making it to MLB varied by nationality. Our results showed that while 13.9% of US-born MiLB players made it to MLB, only 7.5% of the international Latino players did (*p* < 0.001; see Table 1). Although we would have liked to control for performance, we were unable to do so because there is not an overall metric of a player’s performance that makes sense across the levels in baseball. However, playing college baseball in the US is sometimes considered an additional training period for players. So, in additional analyses (described in SOM), we confirmed that nationality matters for highest level achieved and longevity beyond the effects of college attendance.

**Table 1:** Study 1 Means, standard deviations, and percentages by nationality.

Measures	US born players *Mean (SD)*	International Latino players *Mean (SD)*	Statistical analysis results
**Highest level achieved** (Levels 1–7)	4.04 (1.97)	2.86 (2.18)	*t* (48,745) = 61.18, *p* < 0.001, *d* = 0.57
**Longevity** (in years)	6.31 (3.78)	4.50 (4.08)	*t* (48,745) = 49.74, *p* < 0.001, *d* = 0.46
**Made it to Majors** (% of group)	13.9%	7.5%	*χ*^2^ (1, *N* = 48,747) = 460.03, *p* < 0.001

See SOM for the results for other nationalities.

We next examined the effects of ethnicity (White, Black, Hispanic) on player outcomes. The vast majority of the international Latino players in our data self-reported as Hispanic (99.1%), so we could not examine the effects of ethnicity for international Latino players. Therefore, we focused our ethnicity comparisons on the US-born players for whom we had self-reported ethnicity information. Our results showed that White players made it to a significantly higher level than Black players but had a shorter longevity (see Table 2). The US-born Hispanic players had highest level and longevity scores that fell in the middle and were not significantly different from the other groups. Thus, these results indicate that there are some differences in US-born players’ outcomes based on their ethnicity, but the difference between US-born and Latin American players is much larger.

**Table 2:** Study 1 Means and standard deviations for US-born players by ethnicity.

Measures	White players (*n* = 9,713)	Black players (*n* = 956)	Hispanic players (*n* = 736)	Statistical analysis results
Highest level achieved (Levels 1–7)	4.60_a_ (1.99)	4.42_b_ (2.17)	4.50_ab_ (2.12)	*F* (2, 11,402) = 4.35, *p* = 0.013
Longevity (in years)	7.69_a_ (4.12)	8.15_b_ (4.93)	7.80_ab_ (4.48)	*F* (2, 11,402) = 5.39, *p* = 0.005

Means with different subscripts were significantly different at the *p* < 0.05 level according to Fisher’s LSD test.

## 3 Study 2

### 3.1 Method

We created a dataset of coaching staff for all 30 MLB organizations, including all their affiliated MiLB teams, as of Opening Day in 2020.^3^ For each coach, we coded their name, coaching position, current level, and nationality. There were 1,100 coaches that had both their current level and nationality available and thus were included in the dataset. Twenty-eight coaches were not from the United States or a Latin American country and thus were excluded from the dataset, leaving 1,072 coaches (740 US-born coaches, 332 international Latino coaches). Current level was coded using the same 1–7 tier scale utilized for the highest level measure in Study 1.

### 3.2 Results

As predicted, the results of an independent-samples t test revealed that US-born coaches were at a significantly higher level within baseball on average (*M* = 4.79, *SD* = 2.21) compared to international Latino coaches (*M* = 2.95, *SD* = 2.40), *t* (1,070) = 12.29, *p* < 0.001, *d* = 0.81. Importantly, our results held when we accounted for contextual factors such as coaching in the Dominican Summer League (see SOM). In other words, baseball coaches from the US were more likely to be coaching at a higher level than coaches from Latin American countries. One factor that might contribute to these results is that international Latino coaches are less likely to have played in the Majors, an experience that helps coaches obtain higher positions (see SOM).

## 4 Study 3

### 4.1 Method

We surveyed 148 baseball players who were about to begin the 2022 season in the Frontier League, an independent, professional MLB Partner League (MLB.com, 2025). The participants were mostly White Americans (72.4% White, 81.1% from the US).

Players who agreed to complete the survey began by selecting the language they wanted to take the study in (English or Spanish) and then reported information about their demographics and playing backgrounds. Then participants rated themselves on several performance items using a 5-point scale (*1 = poor, 5 = great*) as a measure of their subjective performance (*M* = 3.69, *SD* = 0.78; *α* = 0.85). Next, they rated their agreement with items measuring inclusion (“This team gave me the feeling that I belong;” *α* = 0.97; from the belonging subscale of the Group Inclusion Scale; Jansen et al., 2014), task cohesion (e.g., “Our team was united in trying to reach its goals for performance;” *α* = 0.73; Carron et al., 1985), and social cohesion (“Our team liked to spend time together in the offseason;” *α* = 0.69; Carron et al., 1985) using 5-point Likert scales (*1 = strongly disagree, 5 = strongly agree*). Though our main variable of interest is inclusion, we included measures of cohesion because they are commonly used in sport studies and provide additional information about how players relate to their teammates. See SOM for more information.

### 4.2 Results

For each analysis, we used all the relevant data we had; some participants skipped questions or otherwise did not answer the entire survey. Due to the small number of international Latino players in this dataset, we focused our comparisons on White vs. non-White players (collapsing across non-White ethnicities).

White players reported significantly greater inclusion (*M* = 4.25, *SD* = 1.00) than non-White players (*M* = 3.78, *SD* = 1.29), *t* (105) = 2.02, *p* = 0.046, *d* = 0.43. The inclusion scale was significantly correlated with the mean performance rating, *r* (107) = 0.426, *p* < 0.001. Thus, people who felt like they were more included within the team thought they performed better on the field. We also found significant correlations between inclusion and both task cohesion, *r* (107) = 0.459, *p* < 0.001, and social cohesion, *r* (107) = 0.380, *p* < 0.001. Interestingly, inclusion correlated more strongly with performance, task cohesion, and social cohesion for the non-White players than for the White players (see Table 3). We do not want to make too much of the comparisons due to the small sample size of non-White players, but the big difference for social cohesion is especially interesting (indicating perhaps that minority players can feel included even if they are not socially close to others, whereas White players feel included if they are friends with their teammates).

**Table 3:** Means, standard deviations, and bivariate correlations for variables in Study 3 by player ethnicity.

White players (*n* = 77)				
Variable	*M* (*SD*)	1	2	3
1. Inclusion	4.25 (1.00)			
2. Performance	3.65 (0.79)	0.423*		
3. Task cohesion	3.49 (0.97)	0.420*	0.336*	
4. Social cohesion	3.68 (0.80)	0.190	0.130	0.436*

Non-white players (*n* = 30)				
Variable	*M* (*SD*)	1	2	3
1. Inclusion	3.78 (1.29)			
2. Performance	3.77 (0.75)	0.521*		
3. Task cohesion	3.36 (0.90)	0.570*	0.277	
4. Social cohesion	3.16 (1.05)	0.566*	0.289	0.669*

*p < 0.05.

## 5 Discussion

Across three studies, we examined whether the high diversity on the field in MLB reflects that the pipeline is inclusive, as evidenced by equal opportunities for advancement and player attitudes. In Study 1, we found that international Latino MiLB players are not making it to as high of a level in baseball or having as long of a playing career. In Study 2, we found that international MiLB and MLB Latino coaches were also more likely to be at a lower level in baseball than US-born coaches. Finally, Study 3, we found that non-White players in the Frontier League reported a lower sense of inclusion on their teams. Overall, our studies provide some evidence that despite high player and coach diversity in the Majors, there is still work to be done.

### 5.1 Limitations and future directions

Though our data in Studies 1 and 2 provide evidence that international Latino players and coaches are less likely to progress into higher levels of baseball compared to US-born players and coaches, we are unable to speak to what causes this gap in success. We hope that future research can delve into the reasons for these differences (e.g., language barriers, instances of discrimination). However, our results do clearly indicate that there is still room for improvement at the lower levels of baseball when it comes to making this diverse league truly inclusive, and the current norms for measuring and tracking diversity in sports may be limited. Further, although we cannot be sure of the causal order between inclusion and performance in Study 3, and therefore we cannot determine whether increasing inclusion would increase performance, the results are very interesting nonetheless and should provide some motivation to baseball teams to try to be more inclusive.

Ideally, we would have liked to run the analyses in Studies 1 and 2 controlling for objective performance to rule out that US-born players and coaches advanced higher because they performed better. However, because the players start at different levels in the Minors, and performance statistics change by level (as it is harder to perform well in higher levels), it was not possible to find a performance statistic with the same meaning across players. We also do not know of any performance statistics that indicate coaching ability. Moreover, we would have liked to account for an individual’s native language and years in the US, along with coach race in Study 2, had we possessed the necessary data. As technology advances and more data becomes available, we urge researchers and organizations to develop even more holistic data repositories.

It is important to note that due to the shifts in the meaning of racial and ethnic labels/groups across cultures (e.g., 99.1% of international Latino players in Study 1 self-reported as “Hispanic,” though some may be perceived as “Black” within a US context) and limitations in how demographic information is collected currently (e.g., there are limited ethnicity labels for players to choose from when self-reporting), we were unable to meaningfully examine how nationality and ethnicity intersect with one another in the current context. As organizations make changes in how data is collected, future researchers will be able to examine how multiple salient identities intersect to create unique experiences for players.

### 5.2 Practical implications

As sports become more multicultural and diverse, sports organizations should carefully consider how they utilize DEI metrics. Especially for leagues like MLB where there is salient diversity among many aspects of social identity (e.g., nationality and ethnicity; Zitek et al., 2024), it is important to critically evaluate how data is collected and interpreted. In line with current best practices in research, MLB (and others) should consider asking demographic information as open-ended questions to allow full self-reporting, rather than providing specific categories (e.g., White, Black, Hispanic). If it is unrealistic to allow open-ended questions, the multiple-choice questions should at the very least be updated in two important ways. First, the options provided to select from should be expanded to include relevant racial/ethnic categories from the prominent cultures and countries represented in the data. Second, participants should be able to select multiple racial/ethnic categories, rather than having to choose one or select “two or more,” which does not provide meaningful information for interpretation.

More broadly, to utilize DEI metrics more effectively, organizations should ensure that they collect data in a context-relevant way. Looking specifically at baseball, both ethnicity and nationality are context-relevant (Zitek et al., 2024). By examining the data at the intersections of ethnicity and nationality, we can better understand that while overall there is high representation of “people of color” within baseball at the player and coach level, different minority groups have different experiences. The challenges faced by international Latino players and coaches may not be the same as those faced by US-born Hispanic players and coaches, and thus these issues require different solutions. It is important for the different DEI initiatives to recognize that a “one-size-fits-all” approach does not work, and the programs should be targeted toward different groups (Apfelbaum et al., 2016). Analyzing the data using a context-relevant approach provides more clarity to the causes of inequity, which ultimately paves a better path forward in creating solutions.

In addition to shifting how data is collected, organizations must also carefully consider how data is analyzed and the conclusions drawn from DEI metrics. While the TIDES Report Cards currently provide incredibly valuable information, it is important for individual leagues to examine how well the data represents their sport and what meaning they can take away from these reports. For example, in this paper, we have shown how the interpretation of high player diversity in MLB shifts when we examine progression though the MiLB pipeline and the likelihood of making it to the Majors. By failing to include the Minors in the assessment, the barriers to one of the most vulnerable populations (international Latino players) might be overlooked, which risks perpetuating inequality within baseball.

We hope that our work provides some insight into the importance of triangulating between various DEI metrics before making policy decisions. In this paper, we provide a metric illustrating a disparity between international Latino players and coaches and US-born ones (Studies 1 and 2) and a metric of player inclusion (Study 3). By combining these metrics with the diversity metrics provided in the Report Cards, more accurate and appropriate policy decisions can be made. More specifically, despite the high representation of international Latino players and coaches, MLB should continue to focus efforts on mitigating their barriers to success and ensuring baseball is a fair and inclusive sport for everyone.

Regardless of what DEI initiatives are put into place, we recommend that MLB and teams make participation in these programs optional. Past research has demonstrated backlash against mandatory DEI initiatives (Dobbin and Kalev, 2016) and recent work shows that mandatory initiatives can harm autonomy and prevent people from participating authentically (Malcomb and Zitek, 2024). Even though many players enjoy the diversity of baseball (Malcomb et al., in press), they may want to choose to engage in various inclusion-building programs, rather than being forced.

## 6 Conclusion

We applaud leaders in baseball for thinking about diversity, equity, and inclusion, as evidenced by the various programs put in place by MLB and the individual teams and their engagement with our research team. For example, many MLB teams are now hiring education coordinators and offering language classes in English and Spanish to players in all levels to help reduce the language barrier (Malcomb et al., in press). Further, we applaud the immense work done by the TIDES foundation to increase transparency and accountability in sports organizations on their diversity efforts. We do not see our work as opposed to any of these efforts, nor do we intend to suggest that organizations should not measure diversity. Rather, we believe that our results provide important insights for sports organizations and researchers alike into the importance of cultural context and identity when examining diversity, equity and inclusion. The most prominent diversity metrics used in MLB currently point to the success of representation among players and coaches for “people of color,” particularly international Latinos. Although it may be tempting for MLB to focus more strongly on gender diversity and front office diversity in the coming years, our results suggest that more progress can still be made in the lower levels. Despite the high diversity grades, some baseball players and coaches in the Minors seem to have less ability to advance than others. Specifically, international Latino players do not make it to as high of a level in baseball as US-born players, and international Latino coaches may get stuck in the lower levels of coaching. Moreover, in the independent Frontier League, the non-White players feel less of a sense of inclusion.

Through a deeper analysis of the full pipeline of players and coaches, and by utilizing social identity groups relevant to the context of baseball, we have highlighted a few major limitations with diversity metrics as they are currently used in sports and provide insight into how sports organizations and researchers can better measure and interpret diversity. DEI metrics are a powerful tool that organizations should continue to build and utilize, but organizations should also ensure they spend time understanding what they are measuring, how they are measuring it, and what the limitations of their data are.

## Data Availability

The datasets presented in this article are not readily available to maintain the privacy of identifiable information within the data. Requests to access the datasets should be directed to the corresponding author.

## Footnotes

^1^One additional goal of MLB is to increase the number of African-American players; despite high representation of people of color, African-American representation specifically has been decreasing.

^2^Note that MLB eliminated SS ball in 2021 (Baseball Reference, 2023b), but we otherwise kept the same coding scheme for players in the final two years of our dataset.

^3^Note that there ended up being no 2020 MiLB season due to the pandemic, but we were able to capture the intended coaching staff before decisions to cancel the season were made.
