# Supplementary material for: The illusion of inclusion: examining the limitations of diversity metrics in baseball
Source: Front Psychol. 2025 Mar 26;16:1512033. doi: 10.3389/fpsyg.2025.1512033 (PMC11979116; doi:10.3389/fpsyg.2025.1512033)
Supplement: Supplementary file 1 [file Supplementary_file_1.docx]

**Illusion of Inclusion: Supplemental Online Materials**

A note from the authors: We cannot post our data publicly. Instead, we have provided additional information and analyses in this document to be as transparent as possible. Please contact the authors for more information about the data or our findings.

**Study 1 Additional Information**

**Method**

This study focused on the career progression of players in the MLB system who played at some point in the 1992-2022 range in either the Minors and/or Majors. We collected nationality and ethnicity data as follows from these players.

***Nationality****.* Nationality is taken from the public biographical information collected on the baseball players through Baseball Cube. We focused on international Latino players in Study 1 (and international Latino coaches in Study 2) instead of all international players and coaches for a couple reasons. First, most international players are from Latin American and Caribbean countries (91.4% in our data), with very few players from other countries, making it hard for us to draw any meaningful conclusions from the data on international players outside of Latin America. Further, people within the baseball community tend to talk about groups of players as either the “US players” or the “Latin American players.” This distinction within the baseball community we are studying made it important for us to examine the unique experiences of Latin American players separate from other international players who likely experience different challenges and barriers. Therefore, our primary nationality analyses involved players from the US (*n* = 30,343) or a Latin American country/territory (*n* = 18,404), leaving 48,747 players in the analysis in the main text. Below we report results from the other international players (*n* = 1,730) as an interesting comparison group.

***Ethnicity****.* The ethnicity data we have in the dataset was collected by MLB and is the self-reported ethnicities from the baseball players themselves. Many players do not have ethnicity data available due to the voluntary self-reporting methods used by MLB to acquire the data (note also that there are variations in the amount of ethnicity data recorded by year). In the data we received from MLB, there were the following ethnicities coded: Asian, Black, Hispanic, Native American or Native Alaskan, Native Hawaiian or Other Pacific Islander, White, and two or more races. In our analysis of ethnicity, we only included the following three groups: White, Black, and Hispanic. This decision was made for two reasons. First, these three ethnicities are highly relevant within the context of professional baseball. Second, there were not large enough sample sizes for the other ethnicities to have meaningful results in the analysis. In our full dataset there are 10,204 players who identified as White, 8,213 players who identified as Hispanic, and 1,012 players who identified as Black. There were 388 players who identified as one of the other ethnicities or as multiple ethnicities, and the rest of the data was missing. It is also important to note that ethnicity and racial identities are socially constructed and context-dependent. This point is especially important in our context of baseball, given that how players self-report their own ethnicities may differ fundamentally based on their own cultural contexts. Therefore, we only utilize the ethnicity variable for the US-born players in the main text and include it in an exploratory way, being aware of the limitations of how we can interpret this measure.

**Results**

***Making It Past A Ball***

In the main text we report that international Latino players make it to lower levels of baseball than US-born players. However, this could possibly be driven by a lower starting level for the international Latino players because, for example, the international Latino players are able to sign earlier than the US-born players and are less likely to attend college before joining MiLB. Because some players who played in college start in Class A instead of Rookie Ball, and because Class A is sometimes considered to be the first real year of professional baseball (Moore, 2013), we ran a separate analysis examining whether a player made it past the A level or not. Our results showed that while 41.7% of US-born MiLB players made it past A ball, only 26.2% of the international Latino players did, χ^2^(1, *N* = 48,747) = 1207.30, *p* < .001.

***The Role of College Attendance***

We then wanted to examine how attending college might affect the highest level achieved by players. College players get extra training and often start at higher levels in the Minors, and US-born players are more likely to have attended college (*r* = .77). Thus, we ran a 2 (nationality) X 2 (college attendance) ANOVA predicting highest level achieved. There was a significant main effect of nationality, *F*(1, 48,743) = 117.94, *p* < .001, η^2^ = .002, again showing that US-born players make it to a higher level on average. There was also a significant main effect of college, *F*(1, 48,743) = 240.70, *p* < .001, η^2^ = .005, showing that players who attended college make it to a higher level, as we expected. And finally, there was a significant interaction between nationality and college attendance, *F*(1, 48,743) = 167.44, *p* < .001, η^2^ = .003. As shown in Supplemental Figure 1, attending college led players to make it to a higher level on average for both US-born and international Latino players, but the effect was much larger for the international Latino players. Specifically, the international Latino players who did not attend college (*N* = 17,851) had a highest level that was at least one level lower on average than international Latino players who went to college (*N* = 553), US-born players who did not go to college (*N* = 5,369) and US-born players who did go to college (*N* = 24,974). Thus, it does not seem like attending college accounts for the differences in career outcomes by nationality reported in the main text. US-born players who do not attend college have better outcomes than Latin American players who do not attend college. Nationality is clearly an important factor when predicting success in MiLB.

Although a relatively small percentage of international Latino players play baseball in college, those that do end up making it to a higher level in professional baseball than those that do not. There are a variety of possible reasons why this is the case. For instance, playing in college can help these players develop their skills, the better players are more likely to be recruited for college teams, and players with more resources are able to go to college instead of feeling the pressure to start professional baseball as soon as possible. Whatever the reason, it is clear from the data that international Latino players who do not attend college seem to have additional barriers to career success.

**Supplemental Figure 1. *Highest level obtained (plus 95% confidence intervals) based on nationality and college attendance.***


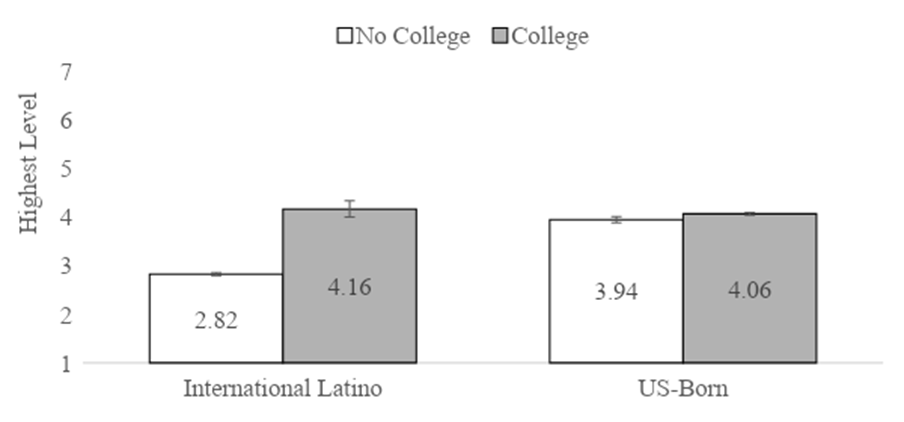


The longevity results showed a similar pattern when accounting for both nationality and college attendance. There was a significant main effect of nationality on longevity, *F*(1, 48,743) = 69.59, *p* < .001, η^2^ = .001, again showing that US-born players play longer on average. There was also a significant main effect of college, *F*(1, 48,743) = 158.55, *p* < .001, η^2^ = .003, showing that players who attended college play longer. And finally, there was a significant interaction between nationality and college attendance, *F*(1, 48,743) = 193.15, *p* < .001, η^2^ = .004, where the international Latino players who did not attend college lasted for shorter (*M* = 4.43, *SD* = 4.03) than the international Latino players who went to college (*M* = 6.78, *SD* = 4.76), the US-born players who did not go to college (*M* = 6.41, *SD* = 4.55), and the US-born players who did go to college (*M* = 6.29, *SD* = 3.59).

Ideally, we would have liked to rerun the highest level and longevity analyses controlling for playing statistics in case the US-born players were making it to a higher level because they were performing better in the Minors than the international Latino players. However, because the players start at different levels in the Minors, and performance statistics change by level (as it is harder to perform well in higher levels), it was not possible to find a performance statistic with the same meaning across players.

***Other International Players***

For an additional analysis, we also examined the highest level obtained by other international players in comparison to the US-born and Latin American players. These other international players came from a variety of countries including Australia, Canada, Japan, and Korea. These other international players had highest level (*M* = 3.62, *SD* = 2.18) and longevity (*M* = 5.74, *SD* = 4.60) statistics that were significantly lower than those of the US-born players (*p* < .001) and significantly higher than those of the international Latino players (*p* < .001). This demonstrates that while there seem to be challenges for all international players, there are particular challenges for the international Latinos.

***Further Exploration of Ethnicity***

We reported our ethnicity results in the main text. Here we add more details about the comparisons across groups. The results of a one-way ANOVA showed that there were small differences in highest level achieved by ethnicity, *F*(2, 11,402) = 4.35, *p* = .013. Specifically, of the US-born players with self-reported ethnicity data, White players made it to a significantly higher level than Black players, *t*(11,402) = 2.72, *p* = .006, *d* = 0.09, and to a slightly (but not significantly) higher level than Hispanic players, *t*(11,402) = 1.35, *p* = .178, *d* = 0.05. The Black and Hispanic players did not differ significantly from each other in highest level, *t*(11,402) = 0.83, *p* = .405, *d* = 0.04. The results were different for longevity. There was again a difference by ethnicity among the US-born players, *F*(2, 11,402) = 5.39, *p* = .005, but White players had significantly shorter longevity in baseball than Black players, *t*(11,402) = 3.25, *p* = .001, *d* = 0.11, and slightly (but not significantly) shorter longevity than Hispanic players, *t*(11,402) = 0.73, *p* = .468, *d* = 0.03. The Black and Hispanic players did not differ significantly from each other, *t*(11,402) = 1.68, *p* = .093, *d* = 0.08.

**Study 2 Additional Information**

**Results**

We first wanted to examine the effect that playing in MLB had on a coach’s outcome. As reported in Study 1 in the main text, Latin American players have a harder time making it to the Majors than US-born players. In a regression predicting coaching from nationality (US-born = 1, Latin American = 0) and whether they played in MLB or not (1 = yes, 0 = no), both variables were significant predictors of the level the coaches were at. Thus, playing at the MLB level seems to be an important factor affecting coaching outcomes in addition to nationality. In fact, including playing at the MLB level in the regression analysis (*b* = 1.03, *t*(1,069) = 6.98, *p* < .001) slightly weakened the effect of nationality (from *b* = 1.84, *t*(1,070) = 12.29, *p* < .001 to *b* = 1.65, *t*(1,069) = 11.04, *p* < .001). Because nationality was correlated with playing at the MLB level in this dataset, *r* = .186, *p* < .001, and in the dataset analyzed in Study 1, it is possible that the difference in coaching outcomes is due in part to the difference in playing outcomes. If Latin Americans are unable to make it to as high of a level as players, it might also prevent them from progressing as coaches given that MLB playing experience is clearly valued.

It is possible that the effect reported in the main text is being driven by coaches from Latin American countries being disproportionately placed in the Rookie-level Dominican Summer League, which is located in the Dominican Republic. To rule out this explanation, we ran the analysis again excluding all coaches at the Rookie Ball level. In this analysis, there were 792 coaches (632 US-born, 160 Latin American). Again, the results of an independent-samples t test revealed that coaches from the United States (*M* = 5.44, *SD* = 1.68) were at a significantly higher level of baseball than coaches from Latin American countries (*M* = 5.05, *SD* = 1.85), *t*(790) = 2.57, *p* = .010, *d* = 0.23. Though the effect is much weaker when excluding coaches at the Rookie level, there is still a significant difference. Finally, we re-ran this analysis (where we excluded the Rookie ball coaches) when accounting for whether the coach had made it to the MLB level as a player. When controlling for playing experience at the MLB level (1 = yes, 0 = no), we found that the effect of nationality weakened further, *b* = 0.28, *t*(789) = 1.89, *p* = .059, and that playing at the MLB level was a stronger and significant predictor of the level of the coach, *b* = 0.73, *t*(789) = 5.88, *p* < .001.

**Study 3 Additional Information**

**Method**

In this study, we worked with Frontier League staff to survey 148 baseball players who were about to begin the 2022 season in the Frontier League, an independent, professional MLB Partner League. This independent league is not a direct feeder league into MLB in the same way that MiLB is, but the coaching staff and organization do try to help players get opportunities in the MLB system. Thus, it is part of the pipeline to the Majors and is still relevant when we examine inclusion in professional baseball in the US.

We had hoped to get a larger sample, but fewer people completed the survey than intended. The participants were mostly White Americans (72.4% White; 81.1% from the US), ranging in age from 20 to 52 (*M_age_* = 25.25, *SD* = 3.67). For sample size reasons, we collapsed ethnicity into two groups. Players were categorized as White if they selected “White” for their race/ethnicity in the survey, and they were categorized as non-White if they selected “Black,” “Hispanic/Latino,” “Asian,” or “Other.” People who selected both “White” and another race/ethnicity were not included in our ethnicity analyses as it was unclear which categorization was more appropriate. Of the 20 respondents who identified as international players, 9 were from Canada, 1 was from the Dominican Republic, 5 were from Puerto Rico, and 5 were from Venezuela). Note that there are fewer international Latino players in the Frontier League than in MiLB and MLB, which changed the focus of some of our analyses. Some of the players who completed this survey had also played in MiLB or MLB in the past (31.7%). Many participants skipped questions or otherwise did not answer the entire survey. For each analysis, we used all of the relevant data we had.

As mentioned in the main text, for this research project, participants responded to questions about their backgrounds, perceived performance (we took the mean of their ratings of the quality of their individual performance, team performance, physical development, mechanical development, development of baseball knowledge, development of approach, and overall development as a player), feelings of inclusion (based on the belonging subscale of the inclusivity scale), and task and social cohesion. In addition to these questions, we included several other questions that were of interest to us and the Frontier League staff, most of which were unrelated to the current research.

In the full survey, in addition to what we already mentioned, participants were also asked to think of the team they spent the most time with in 2021 (Frontier League, other independent league, MiLB, or college/amateur). They then responded to a variety of questions about their manager and an assistant coach and how these coaches interacted with them. Participants also responded to some questions about their own potential to coach, including, “Are you interested in coaching after you finish playing?” (1 = *definitely not*, 5 = *definitely yes*) and “How fair do you think the process is for getting a coaching position?” (1 = *extremely unfair*, 5 = *extremely fair*). We followed up with an open-ended item where participants could tell us why they thought the process of becoming a coach was or was not fair.

**Results**

We examined how inclusion (specifically a sense of belonging) varied across players of different races and nationalities. For sample size reasons, we had to collapse across races other than White and nationalities other than American. As reported in the main text, the results of an independent-samples t-test showed that White baseball players reported a significantly greater sense of belonging (*M* = 4.25, *SD* = 1.00) than non-White players (*M* = 3.78, *SD* = 1.29), *t*(105) = 2.02, *p* = .046, *d* = 0.43. We did not find a significant difference between the players born in the United States (*M* = 4.15, *SD* = 1.04) as compared to other countries (*M* = 4.18, *SD* = 0.88), *t*(77) = 0.09, *p* = .929, *d* = 0.03, but the number of international players was very small (many players skipped the nationality question). Thus, these results provide some evidence that the non-White players in the Frontier League feel a lower sense of belonging, but we were unable to determine whether the Latin American players were less likely to feel like they belong due to the small number of Latin American participants.

We also examined responses to the questions about coaching in the future. We originally asked these questions to try to answer some remaining questions from Study 2 about whether players from different nationalities (or ethnicities) differ in their interest in coaching and/or perceptions that the process of getting a coaching job is fair. However, due to the small sample sizes of non-White players and international players, we again had to interpret our results very cautiously. The results of independent-samples t tests showed that White baseball players, compared to the non-White players, reported slightly less interest in coaching (*M* = 3.60, *SD* = 1.11; vs. *M* = 3.71; *SD* = 1.10) and perceived that the process was slightly more fair (*M* = 3.29, *SD* = 0.89; vs. *M* = 3.17; *SD* = 0.79), *t*(130) = 0.53, *p* = .596, *d* = 0.10; *t*(129) = 0.70, *p* = .483, *d* = 0.14. Moreover, US-born baseball players, compared to the international players, reported basically the same interest in coaching (*M* = 3.60, *SD* = 1.14; vs. *M* = 3.58; *SD* = 1.07) and perceived that the process was slightly more fair (*M* = 3.36, *SD* = 0.84; vs. *M* = 3.00; *SD* = 0.88), *t*(95) = 0.08, *p* = .935, *d* = 0.02; *t*(95) = 1.66, *p* = .100, *d* = 0.42. None of these differences were significant, but given the pattern of means, it seems that the White and US-born players do not have a greater interest in coaching, but they do think the process is slightly fairer. Our largest effect was the comparison between the US-born and international players, where the international players rated the process of getting a coaching position as less fair (*p* = .1). Again, we do not want to make too much of the results, but the differences in perceived fairness do seem to match up with the inequities we saw in Study 2. In the open-ended question, many players, including White and US-born players, mentioned that getting a coaching position was dependent on their connections. Players said things such as, “Like in any job it is political,” “Its [*sic*] all about who you know,” and “It's all about connections so if you have them it's easy but if you don't it's tuff [*sic*].” Of course, it is possible that if the decision makers are White and US-born, that will make it especially hard for non-White and international players to use their connections.
